# Identification of missing concepts in biomedical terminologies using sequence-based formal concept analysis

**DOI:** 10.1186/s12911-021-01592-w

**Published:** 2021-11-09

**Authors:** Fengbo Zheng, Rashmie Abeysinghe, Licong Cui

**Affiliations:** 1 Department of Computer Science, University of Kentucky, Lexington, KY USA; 2 School of Biomedical Informatics, University of Texas Health Science Center at Houston, Houston, TX USA; 3 Department of Neurology, McGovern School of Medicine, University of Texas Health Science Center at Houston, Houston, TX USA

**Keywords:** Quality assurance, Concept enrichment, Formal concept analysis, SNOMED CT, NCI Thesaurus

## Abstract

**Background:**

As biomedical knowledge is rapidly evolving, concept enrichment of biomedical terminologies is an active research area involving automatic identification of missing or new concepts. Previously, we prototyped a lexical-based formal concept analysis (FCA) approach in which concepts were derived by intersecting bags of words, to identify potentially missing concepts in the National Cancer Institute (NCI) Thesaurus. However, this prototype did not handle concept naming and positioning. In this paper, we introduce a sequenced-based FCA approach to identify potentially missing concepts, supporting concept naming and positioning.

**Methods:**

We consider the concept name sequences as FCA attributes to construct the formal context. The concept-forming process is performed by computing the longest common substrings of concept name sequences. After new concepts are formalized, we further predict their potential positions in the original hierarchy by identifying their supertypes and subtypes from original concepts. Automated validation via external terminologies in the Unified Medical Language System (UMLS) and biomedical literature in PubMed is performed to evaluate the effectiveness of our approach.

**Results:**

We applied our sequenced-based FCA approach to all the sub-hierarchies under *Disease or Disorder* in the NCI Thesaurus (19.08d version) and five sub-hierarchies under *Clinical Finding* and *Procedure* in the SNOMED CT (US Edition, March 2020 release). In total, 1397 potentially missing concepts were identified in the NCI Thesaurus and 7223 in the SNOMED CT. For NCI Thesaurus, 85 potentially missing concepts were found in external terminologies and 315 of the remaining 1312 appeared in biomedical literature. For SNOMED CT, 576 were found in external terminologies and 1159 out of the remaining 6647 were found in biomedical literature.

**Conclusion:**

Our sequence-based FCA approach has shown the promise for identifying potentially missing concepts in biomedical terminologies.

## Background

Biomedical terminologies or ontologies have played important roles in various biomedical research and applications, including data annotation, data integration, data sharing and exchange, natural language processing (NLP), and clinical decision support [1–3]. For instance, BioPortal [4–6], the world’s most comprehensive repository of biomedical terminologies, contains over 800 terminologies that have been used to support a wide spectrum of scientific projects in biomedicine.

Biomedical terminologies are constantly evolving due to the growing knowledge in biomedicine, new requirements from emerging biomedical applications, and the progressive nature of terminology development [7, 8]. Therefore, terminology management always involves the addition of new concepts along with their definitions, as well as deprecation and deactivation of obsolete ones. For example, SNOMED CT is released regularly every six months [9]. In the January 2019 release of SNOMED CT (International Edition), 11,903 new concepts were added and 3035 concepts were deactivated. For the National Cancer Institute (NCI) Thesaurus, it is updated every month with an average of roughly 700 new concepts added in each release [10].

To keep pace with the rapidly evolving biomedical knowledge, researchers have paid particular attention to the automatic identification of missing or new concepts for biomedical terminologies (so-called *concept enrichment*). In general, there are mainly two types of approaches for concept enrichment in a terminology: (1) importing concepts from external knowledge such as another terminology [11–14]; (2) utilizing the intrinsic knowledge within the terminology itself [15–17].

In a recent work [18], we prototyped a lexical-based Formal Concept Analysis (FCA) approach that leverages intrinsic knowledge to identify potentially missing concepts in the NCI Thesaurus. In [18], the words appearing in concept names were taken as FCA attributes to generate new concepts in the form of bags of words. However, such bags of words are unordered, leaving the question of how to precisely naming the concepts based on bags of words open. Moreover, it remains unsolved where the potentially missing concepts should locate.

To address the concept naming and positioning barriers, in this paper, we introduce a sequence-based FCA approach to identifying potentially missing concepts in a given terminology. We leverage the concept names as FCA attributes to construct the formal context, i.e., for each concept, its sole attribute is the sequence of its own name. FCA formal concepts are obtained by finding the longest common substrings between sequences so that the newly derived concepts can be directly named by their FCA attributes. We further investigate the “subconcept-superconcept” relations between newly formalized concepts and original concepts to suggest the positions where the potentially missing concepts could be added. To evaluate our approach in an automatic way, we leverage extrinsic knowledge in the Unified Medical Language System (UMLS) and biomedical literature in PubMed to validate the potentially missing concepts identified.

### Formal concept analysis

FCA, a mathematical theory for concept formalization, can derive a concept hierarchy from a collection of objects and attributes [19]. The input of FCA is *formal context* $K = (O, A, R)$, where *O* is a set of objects, *A* is a set of attributes, and *R* is a binary relation between *O* and *A*. Conventionally, to generate new concepts, we need two kinds of operators—derivation operators $\uparrow : 2^O \rightarrow 2^A$ and concept-forming operators $\downarrow : 2^A \rightarrow 2^O$. The operators are defined, for each $X \subseteq O$ and $Y \subseteq A$, as follows:

$$\begin{aligned} X^\uparrow= & {} \{a\in A|\forall o \in X\!\!:(o,a) \in R\},\\ Y^\downarrow= & {} \{o\in O|\forall a \in Y\!\!:(o,a) \in R\}, \end{aligned}$$

where (*o*, *a*) $\in R$ means that object *o* has attribute *a*. In other words, $X^\uparrow$ is the set of all attributes shared by all objects in *X*, and $Y^\downarrow$ is the set of all objects sharing all attributes in *Y*.

A *formal concept* of *K* is a pair (*X*, *Y*) with $X \subseteq O$ and $Y \subseteq A$ such that $X^\uparrow = Y$ and $Y^\downarrow = X$. Given two formal concepts $(X_1,Y_1)$ and $(X_2,Y_2)$, they form a subconcept-superconcept relation $(X_1,Y_1)\le (X_2,Y_2)$ iff $X_1 \subseteq X_2$ (or $Y_2 \subseteq Y_1$). All formal concepts derived from the formal context *K* together with the subconcept-superconcept relations form a complete lattice, where lattice is a desired property for ontologies [20, 21].

### Extrinsic knowledge for validation

In this work, we leverage external terminologies in the UMLS and biomedical literature in PubMed to evaluate the effectiveness of our approach.

#### Unified medical language system

The UMLS, developed by the US National Library of Medicine, integrates various health and biomedical vocabularies and standards to enable interoperability between different applications and systems [22, 23]. It has been used in supporting a wide range of applications in biomedicine including information retrieval, NLP, phenotyping, quality assurance, and clinical decision support [24–28].

The UMLS consists of three knowledge sources: the Metathesaurus that contains concepts from many terminologies, the Semantic Network that contains semantic types and their relationships, and the SPECIALIST Lexicon and Lexical Tools to facilitate NLP [29].

In the Metathesaurus of UMLS, different terms from various terminologies with the same clinical or health meaning are mapped to a concept and assigned a concept unique identifier (CUI). For example, “Myocardial Infarction,” “Infarction of heart,” “Heart attack,” and “Cardiovascular stroke” from different source terminologies represent the same meaning and are assigned a unique CUI *C0027051*. Each UMLS concept (CUI) is assigned at least one semantic type in order to provide a consistent categorization of all concepts. For example, concept “Myocardial Infarction” (CUI *C0027051*) is assigned a semantic type “Disease or Syndrome.”

#### Biomedical literature in PubMed

PubMed is a database of bibliographic information drawn primarily from the life sciences literature. It compromises more than 32 million records representing articles in the biomedical literature. In general, a basic bibliographic citation provides the title of the article, abstract published with the article, and controlled vocabulary search terms [30].

## Methods

In this work, we develop a sequence-based FCA approach to detect potentially missing concepts in the NCI Thesaurus and SNOMED CT to enhance their completeness of concept coverage. There are mainly three steps: (1) pre-process concept names; (2) use processed sequences to construct formal context and derive new concepts by sequence-based intersection (i.e., perform sequence-based FCA); and (3) given a newly formalized concept, identify original concepts that potentially serve as its subtype(s) and supertype(s) for concept positioning.

### Pre-processing

To create a more robust formal context, we pre-process concept names in two steps. We first normalize words appearing in concept names using LuiNorm [31], a lexical tool provided by the UMLS. For instance, “arteries” is normalized to “artery.” Secondly, we maintain mappings between single-word preferred names and their single-word synonyms. If a concept name contains a word that serves as a synonym, we replace that word with its corresponding single-word preferred name. For example, in the NCI Thesaurus, the appearance of “before” in concept names will be substituted by its preferred name “prior.” This two-step pre-processing converts words with variations or synonyms to a unified appearance and benefits the concept-forming process in the FCA.

### Sequence-based formal concept analysis

**Fig. 1:**
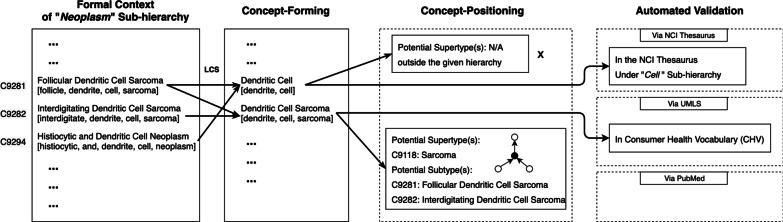
An example of detecting potentially missing concepts in Neoplasm sub-hierarchy in the NCI Thesaurus, showing the pipeline of our sequence-based FCA approach. The longest common substring “Dendritic Cell” generated from Concept “Follicular Dendritic Cell Sarcoma” and “Histiocytic and Dendritic Cell Neoplasm” does not have any existing supertype and thus considered as “outside the given hierarchy.” A qualified missing concept “Dendritic Cell Sarcoma” derived from concept “Follicular Dendritic Cell Sarcoma” and “Interdigitating Dendritic Cell Sarcoma” appears in the Consumer Health Vocabulary (CHV) in the UMLS

While detecting potentially missing concepts in a given hierarchy of concepts, we consider all concepts in the hierarchy as FCA objects and use the pre-processed concept name sequences as FCA attributes to construct the formal context. Formally, given a concept *X*, its FCA attribute is a sequence of words $S_X = [XW_1, XW_2, XW_3,\ldots , XW_n]$, where *n* is the length of its name (or the number of words in its name). Figure 1 shows the entire process of our sequence-based FCA approach using an example in which concept completeness of *Neoplasm* sub-hierarchy in the NCI Thesaurus is audited. For example, in the first step where the formal context is formed, the FCA attribute of concept “Follicular Dendritic Cell Sarcoma” is a sequence: [follicle, dendrite, cell, sarcoma].

In our previous work [18], we leveraged a faster multistage concept analysis technique [32] to iteratively derive formal concepts: compute the shared attributes of two concepts to reveal a concept; and perform the pairwise intersection among all the cumulated concepts to reveal a complete list of formal concepts.

In this work, we adopt a similar strategy. Since the formal context is now constructed by sequences, the intersection operation of finding shared attributes of two concepts is re-defined as computing the longest common substring(s) between two sequences. Formal definitions are provided as follows. Given two sequences $S_A=[AW_1, AW_2, AW_3,\ldots , AW_i]$ (length equals to *i*) and $S_B = [BW_1, BW_2, BW_3,\ldots , BW_j]$ (length equals to *j*), we say $S_A$ is a sublist of $S_B$, if there exists *n* ($1<n<j$) such that $[BW_n, BW_{n+1}, BW_{n+2},\ldots , BW_{n+i-1}]$ equals to $S_A$. The longest common substring(s) $S_{LCS}$ of two sequences $S_X$ and $S_Y$ refers to the longest string that is a substring of both $S_X$ and $S_Y$.

While performing FCA, we apply the reformulated intersection to pairs of sequences that represent the FCA formal concepts. The initial set includes sequences of all the original concepts. In the first iteration, we compute the longest common substring of each pair of sequences in the initial set; and we add it into the initial set if the sequence is not included in the initial set. We repeat this sequence-based pairwise intersection until no new sequences (i.e., formal concepts) can be derived. An advantage of using the longest common substrings as shared attributes is that the newly derived formal concepts can be named directly by the generated sequences. For example, in Fig. 1, the longest common substring of [follicle, dendrite, cell, sarcoma] and [interdigitate, dendrite, cell, sarcoma] is [dendrite, cell, sarcoma], which is not included in the original formal context. Therefore, a potentially missing concept with name sequence [dendrite, cell, sarcoma] (i.e., “*Dendritic Cell Sarcoma*”) can be identified.

### Concept positioning

Besides identifying potentially missing concepts, we also predict the position where a missing concept can be placed by investigating the subconcept-superconcept relations between newly derived concepts and the original concepts.

Since the “subconcept-superconcept” relations between formal concepts are derived from lexical features, they may be different from the hierarchical IS-A relations in the original terminology that is organized according to semantic meanings. Therefore, a newly formalized concept may describe knowledge from a branch that is different from the one the original concepts belong to. We call this an “*outside the given hierarchy*” issue. For instance, when detecting potentially missing concepts for *Neoplasm* sub-hierarchy in the NCI Thesaurus, intersecting FCA word attributes of concept “chest wall sarcoma” and concept “chest wall lymphoma” will result in a new concept “chest wall.” However, “chest wall” refers to a part of the body rather than a neoplastic disorder, thus outside the given hierarchy *Neoplasm*. In such cases, even though the newly formalized concept is valid, it should not be added to the audited hierarchy due to the different knowledge branches.

In our sequence-based FCA approach, concept *X* and concept *Y* form a subconcept-superconcept relation (i.e., *X* is a subtype of *Y*) if $S_Y$ is a sublist of $S_X$. Given a potentially missing concept *X*, we retrieve the original concepts that could serve as its subtypes and supertypes in order to pinpoint its potential location in the hierarchy as follows. We first look for *X*’s supertypes. If *X* does not have any supertypes (i.e., there is no existing concept whose name sequence is a sublist of *X*’s), then we consider *X* outside the given hierarchy, because having no supertype (in terms of sequence) often indicates that this concept is likely to represent knowledge that falls in another branch. We call *X* is *qualified* if it has at least one supertype. If *X* is qualified, then we further retrieve its subtypes, i.e., all existing concepts such that $S_X$ is a sublist of their respective sequences. If a missing concept has multiple subtypes, we only retain the most general ones.

Consider the example in Fig. 1, no supertype is identified for newly formalized concept “Dendritic Cell.” Therefore, it is regarded as outside the given hierarchy, and will be removed from the result of potentially missing concepts. Note that “Dendritic Cell” is an existing concept in the NCI Thesaurus but locates in *Cell* sub-hierarchy, which is in accordance with our assumption. In the actual implementation of our approach, we also check if a newly generated concept is existing in the audited terminology (e.g., a synonym for an existing concept in the same sub-hierarchy or included in another sub-hierarchy) and ensure the removal of such cases from the resulting list of potentially missing concepts. When it comes to another newly formalized concept “Dendritic Cell Sarcoma,” it has a supertype “Sarcoma” (C9118) in the same sub-hierarchy. Further computing of its subtypes yields “Follicular Dendritic Cell Sarcoma” (C9281), “Interdigitating Dendritic Cell Sarcoma” (C9282), “Inflammatory Pseudotumor-Like Follicular/Fibroblastic Dendritic Cell Sarcoma” (C150704), and “Thyroid Gland Follicular Dendritic Cell Sarcoma” (C156408). However, “Thyroid Gland Follicular Dendritic Cell Sarcoma” is a subtype of “Follicular Dendritic Cell Sarcoma”, thus removed. After removing the more specific ones, the most general subtypes remaining are “Follicular Dendritic Cell Sarcoma” (C9281) and “Interdigitating Dendritic Cell Sarcoma” (C9282). If “Dendritic Cell Sarcoma” is accepted as a new concept, it is likely to serve as the parent of these two most general subtypes.

**Fig. 2:**
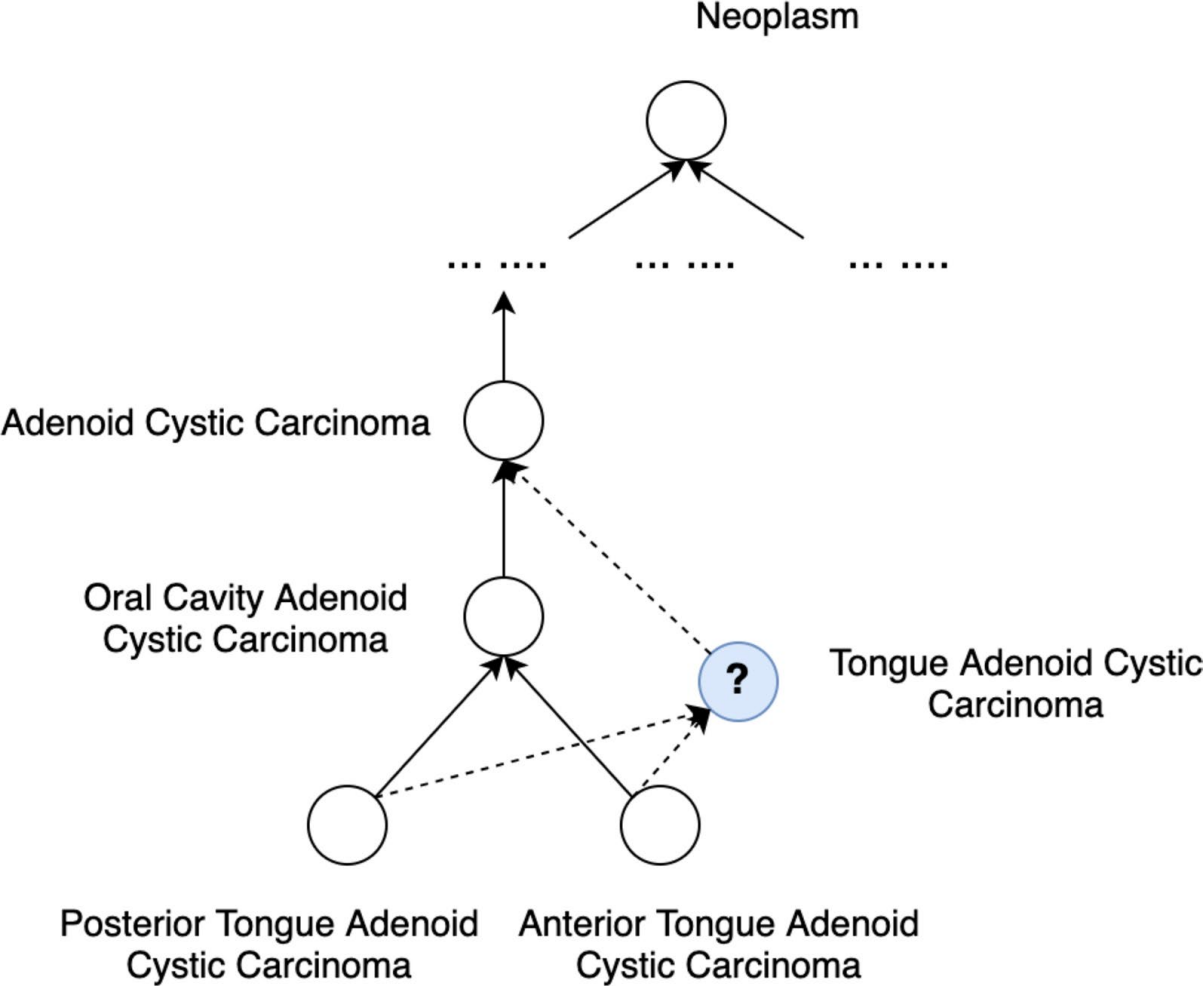
An example of suggesting positions where potentially missing concepts could be added

Figure 2 shows another example, where a potentially missing concept “Tongue Adenoid Cystic Carcinoma” could be located. Its most specific supertype is “Adenoid Cystic Carcinoma” and its most general subtypes are “Posterior Tongue Adenoid Cystic Carcinoma” and “Anterior Tongue Adenoid Cystic Carcinoma.”

**Fig. 3:**
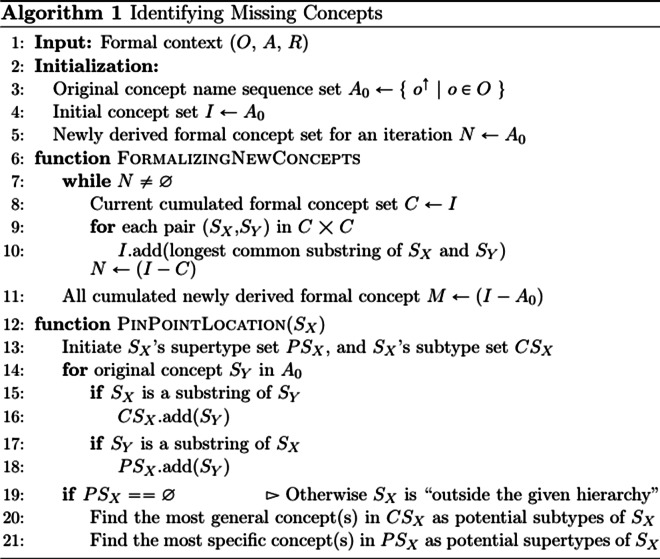
Pseudocode of identifying potentially missing concepts and pinpointing where they may be inserted. Function *FormalizingNewConcepts* shows the multistage intersection and function *PinPointLocation* presents how we compute potential subtype(s) and supertype(s) for potentially missing concepts

Figure 3 presents the pseudocode of the entire process for identifying potentially missing concepts via multistage Formal Concept Analysis (see function *FormalizingNewConcepts*), as well as pinpointing the location where a potentially missing concept may be inserted via computing the concept’s potential subtype(s) and supertype(s) (see function *PinPointLocation*).

### Evaluation

After potentially missing concepts are identified, we validate them via extrinsic knowledge from external terminologies in the UMLS and biomedical literature in PubMed.

#### Validation via external terminologies

For each potentially missing concept identified, we check whether its attribute (i.e., name sequence) appears in any external terminologies in the UMLS. If so, we further look for supporting evidence regarding its positioning. Given a missing concept *X*, we map its subtypes and itself to UMLS concepts (e.g., *X* and its subtype *Y* are mapped to $C_1$ and $C_2$ respectively). For each suggested subsumption relation (e.g., *Y* IS-A *X*), we check if there exists a path *p* between their mapped CUIs (e.g., $C_2$ and $C_1$) in the UMLS such that *p* = $C_2, C_{i1}, C_{i2}, \ldots , C_{ik}, C_1$ such that $C_2$ *IS-A* $C_{i1}, C_{i1}$ *IS-A* $C_{i2}$, ..., and $C_{ik}$ *IS-A* $C_1$. If so, we say that there is a piece of evidence in the UMLS supporting the suggested concept location. In this work, the subtype relations along the path may be from different terminologies.

#### Validation via biomedical literature

If a potentially missing concept is not covered by the UMLS, we further perform a PubMed-based literature search to see whether it exists in biomedical literature. We use the 2020 Production Year MEDLINE/PubMed baseline files, which contains data about over 30 million publications [33]. The enormity of the number of publications needed to be searched, makes it a difficult task to perform a serial search to extract the abstracts containing potentially missing concepts identified in this work. Therefore, we perform an indexed search using Apache Lucene to address this issue [34]. First, we index the titles and abstracts of publications parsed from the XML files in the above-mentioned MEDLINE/PubMed release. Then, given a potentially missing concept *A*, we search the index to extract the publications that contain *A*. For each publication in the query result, we further check if it contains other existing concept names in the terminology (or *A*’s potential subtypes) that include *A* as a substring. If so, that publication will be removed from the query result. To some extent this ensures that the phrase in the query result (i.e., the supporting evidence we found) is for the potentially missing concept itself, rather than for other existing concepts that are more specific (i.e., its potential subtypes). For example, given a potentially missing concept with name “Adenoma With Severe Dysplasia,” the qualified publications are those containing exactly “Adenoma With Severe Dysplasia,” but not its potential subtypes such as “Colorectal Adenoma with Severe Dysplasia,” “Rectal Adenoma with Severe Dysplasia” and “Colon Adenoma with Severe Dysplasia.” Regarding the given example, a qualified publication we found is #12626909 in PubMed, and the relevant sentence is “Of the adenomas, 29 were tubulous, 118 tubulovillous, and 20 villous; adenoma with severe dysplasia was found in 49 cases.”

## Results

We applied our sequence-based FCA approach to all the sub-hierarchies under “Disease or Disorder” in the 19.08d version of NCI Thesaurus and 5 sub-hierarchies in the March 2020 release of SNOMED CT (US Edition), including *Neoplasm and/or hamartoma* (399981008), *Traumatic AND/OR non-traumatic injury* (417163006), and *Degenerative disorder* (362975008) under “Clinical Finding,” as well as *Surgical procedure* (387713003) and *Removal* (118292001) under “Procedure.” In total, 1397 potentially missing concepts were identified in the NCI Thesaurus sub-hierarchies and 7223 in the SNOMED CT sub-hierarchies. Since a concept may belong to different sub-hierarchies (e.g., “Lung Carcinoma” (C4878) belongs to two sub-hierarchies: *Neoplasm* and *Disorder by Site*), we removed the newly formalized concepts that are redundant while calculating the total numbers.

Tables 1 and 2 show the numbers of existing concepts, qualified newly generated concepts, and potentially missing concepts for the audited sub-hierarchies in the NCI Thesaurus and SNOMED CT, respectively. For instance, 3172 new concepts with supertype were derived in the *Surgical procedure* sub-hierarchy of SNOMED CT (see Table 2), among which 108 are included in the SNOMED CT (e.g., synonyms of existing concepts or in other sub-hierarchies) and the remaining 3064 are considered potentially missing.

**Table 1:** The numbers of existing concepts, qualified newly generated concepts, potentially missing concepts, and missing concepts validated via UMLS, concept position supporting evidence found and missing concepts validated via PubMed for each sub-hierarchy under “Disease or Disorder” in the NCI Thesaurus

Sub-hierarchy	# of Existing concepts	# of Newly formalized concepts				
		# of Qualified newly formalized concepts	# of Potentially missing concepts	# of Validated via UMLS	# of Position support in UMLS	# of Validated via PubMed
C35470: Behavioral Disorder	49	4	2	0	0	0
C8278: Cancer-Related Condition	578	43	41	1	1	7
C27551: Disorder by Site	13,595	984	900	32	12	123
C3101: Genetic Disorder	159	8	8	0	0	7
C3075: Hamartoma	63	4	4	0	0	3
C3113: Hyperplasia	81	7	6	1	1	4
C3262: Neoplasm	10,996	1355	1199	46	17	222
C53529: Non-Neoplastic Disorder	4198	119	112	22	7	43
C89328: Pediatric Disorder	528	23	15	0	0	3
C3340: Polyp	110	5	4	2	0	1
C2893: Psychiatric Disorder	231	4	4	1	1	0
C4873: Rare Disorder	915	21	21	3	2	13
C28193: Syndrome	907	68	65	10	4	42

**Table 2:** The numbers of existing concepts, qualified newly generated concepts, potentially missing concepts, and missing concepts validated via UMLS, concept position supporting evidence found and missing concepts validated via PubMed for 5 sub-hierarchy under “Clinical Finding” and “Procedure” in the SNOMED CT

Sub-hierarchy	# of Existing concepts	# of Newly formalzied concepts				
		# of Qualified newly formalized concepts	# of Potentially missing concepts	# of Validated via UMLS	# of Position support in UMLS	# of Validated via PubMed
399981008: Neoplasm and/or hamartoma	8559	953	916	268	239	138
417163006: Traumatic AND/OR non-traumatic injury	12,145	2065	2002	130	81	103
362975008: Degenerative disorder	3286	318	310	30	18	103
387713003: Surgical procedure	20,155	3172	3064	86	44	607
118292001: Removal	9959	2067	1983	107	54	455

### Validation via the UMLS

Tables 1 and 2 also show the number of potentially missing concepts in the NCI Thesaurus and SNOMED CT that appear in (or validated via) external terminologies in the UMLS. In total, 85 potentially missing concepts were validated for the NCI Thesaurus sub-hierarchies, and 576 for the SNOMED CT sub-hierarchies. Table 3 lists 10 examples of validated ones (5 from NCI Thesaurus and 5 from SNOMED CT) and the external terminologies that include them. For example, “congenital muscular dystrophy” derived from our sequence-based intersection between “Merosin-Deficient Congenital Muscular Dystrophy Type 1A” (C118783) and “Ullrich Congenital Muscular Dystrophy” (C123438) is an active concept in terminologies such as Online Mendelian Inheritance in Man (OMIM), Human Phenotype Ontology (HPO) and Consumer Health Vocabulary (CHV).

**Table 3:** Ten examples of missing concepts that appear in the external terminologies in the UMLS

Audited terminology	Potentially missing concept	Supporting external terminolgoy
NCI Thesaurus	Carcinoma with osteoclast-like giant cells	SNOMED CT, MEDCINE
NCI Thesaurus	Peripheral nerve sheath neoplasm	MSH, CHV
NCI Thesaurus	Congenital muscular dystrophy	RCD, OMIM, HPO, CHV, HGNC
NCI Thesaurus	Motor neuropathy	SNMI, OMIM, CHV
NCI Thesaurus	Dyserythropoietic anemia	CHV, MEDCIN, OMIM, HPO
SNOMED CT	Chondrocalcinosis of elbow	MEDCINE
SNOMED CT	Hereditary cerebral amyloid angiopathy	MSH
SNOMED CT	Metatarsal osteotomies	CHV, MEDCIN
SNOMED CT	Removal of foreign body from rectum	MDR
SNOMED CT	Open reduction of fracture of talus	ICD10AM, CPT

The second last column of Tables 1 and 2 shows the number of concept location suggestions supported by subsumption relations between CUIs in the UMLS. For instance, we found a potentially missing concept “hemiplegic migraine” in the NCI Thesaurus that has a potential subtype “Familial Hemiplegic Migraine” (C117009). A path between their mapped CUIs in the UMLS (i.e., CUI C0338484 for “Familial Hemiplegic Migraine” and CUI C0270862 for “hemiplegic migraine”) can be found and the supporting evidence comes from the SNOMED CT (US Edition).

### Validation via biomedical literature

Tables 1 and 2 additionally show the number of potentially missing concepts that can be validated through biomedical literature. In total, 315 potentially missing concepts can be validated for the NCI Thesaurus and 1159 for the SNOMED CT. For example, potentially missing concept “adenoma with severe dysplasia” appears in the abstract of [35] in the sentence “Of the adenomas, 29 were tubulous, 118 tubulovillous, and 20 villous; adenoma with severe dysplasia was found in 49 cases.” Table 4 shows additional five examples of potentially missing concepts that appear in the abstracts of biomedical literature in PubMed.

**Table 4:** Five examples of missing concepts that appear in the abstracts of biomedical literature in MDELINE/PubMed

Potentially missing concept	PMID	Sentence that contains potentially missing concept as entity
Micropapillary breast carcinoma	24362476	Micropapillary breast carcinoma has been recognized as a morphologically and biologically distinct form of breast carcinoma
Composite ganglioneuroblastoma	8108298	We analyzed a composite ganglioneuroblastoma for N-myc copy number at initial resection and 2 years later after progressive disease
Autosomal recessive muscular dystrophy	8202529	We have examined M-laminin expression in mice with autosomal recessive muscular dystrophy caused by the mutation dy
Transcervical excision	28695764	Where possible, cysts should be completely excised, and there is growing evidence that a transoral approach is superior to transcervical excision for nearly all cysts
Root caries lesion	2640753	The root caries lesion was found in 75% of patients

One thing is that some potentially missing concepts could be incorrectly verified. For instance, “lentiginous melanoma” appears in the abstract of [36] in the sentence “We report a case of oral lentiginous melanoma in a Japanese-American man who survived disease-free for more than 5 years after surgery, radiation therapy, and chemotherapy but developed chronic mucositis of the palate under the denture in the primary radiated field.” In this case, the concept is actually “oral lentiginous melanoma” of which “lentiginous melanoma” is a substring. Even though we ensure that the extracted publications do not contain a more specific concept in the audited terminology of which the potentially missing concept is a substring, we cannot guarantee the removal of the cases in which the concepts are not included in the audited terminology (e.g., “oral lentiginous melanoma” is not included in the NCI Thesaurus).

## Discussion

### Outside the given hierarchy issue

In this work, to improve the precision of suggested missing concepts for a hierarchy, we attempt to avoid the “outside the given hierarchy” issues by checking that for a newly formalized concept, whether there exists an original concept in the hierarchy that is more general than it in terms of the sequence (i.e., it has a supertype).

To evaluate the effectiveness of this strategy, we compare the performance of our approach with and without concept positioning enhancement. We leverage the original hierarchical IS-A relations from the audited terminology (i.e., NCI Thesaurus/SNOMED CT) and semantic types from the UMLS that categorize concepts based on their semantic meanings to determine whether a newly derived concept is outside the given hierarchy or not. More specifically, if a newly derived concept can be found in the audited terminology, we will check if it is a synonym of a concept inside the same sub-hierarchy or it falls in another sub-hierarchy (i.e., outside the given hierarchy). In the other aspect, given a potentially missing concept that appears in external terminologies in the UMLS, we map its subtypes and itself to CUIs, and further check if their mapped concepts (i.e., CUIs) share any semantic types. To some extent this could help us decide whether they are representing knowledge in the same branch. If no semantic type is shared, the potentially missing concept is considered outside the given hierarchy.

Table 5 shows the performance differences. For a concept that has a supertype, if it is not outside the given hierarchy, it is considered as a true positive (TP) case; otherwise, it is a false positive (FP) case. Similarly, for the concept that has no supertype, if it is outside the given hierarchy, it is considered as a true negative (TN) case; otherwise, it is a false negative case. For example, while detecting potentially missing concepts for “Surgical procedure” sub-hierarchy in the SNOMED CT, 14,775 concepts were newly formalized from the formal context, out of which 11,603 have no supertype. For newly derived concepts that were included in the SNOMED CT, 97 are true positives while 11 concepts are with supertype but outside the given hierarchy. The precision of adopting concept position enhancement is 89.81% (TP/(TP + FP)). If the enhancement is not employed, all the newly formalized concepts covered by SNOMED CT are considered positive (TP + FP + TN + FN = 2306), however, 1942 (FP + TN) of them are outside the given hierarchy. In this case, the precision is only 15.78%. When it comes to the remaining concepts that appear in the UMLS, there are 86 concepts that have supertypes, among which 82 share semantic types with their subtypes. The precision is then 95.35%. Without the enhancement, all 968 (TP + FP + TN + FN) concepts will be considered positive while only 314 (TP + FN) are potentially representing knowledge in the same branch, leading the precision to become much lower (i.e., 32.44%). It can be seen from Table 5 that without inspecting the existence of supertype, many false positive cases will be suggested while enriching a hierarchy of concepts.

**Table 5:** The performance of our sequence-based FCA approach with and without concept positioning enhancement

Sub-hierarchy	# of All newly formalized concepts	# of Concepts having supertype	# of Newly formalized concepts included in NCIt/SNOMED CT						# of Remaining newly formalized concepts included in UMLS					
			TP	FP	TN	FN	Precison with concept positioning enhancement (%)	Precison without concept positioning enhancement (%)	TP	FP	TN	FN	Precison with concept positioning enhancement (%)	Precison without concept positioning enhancement (%)
C3262: Neoplasm	4338	1355	155	1	646	110	99.36	29.06	44	2	122	21	95.65	34.39
C27551: Disorder by Site	5079	984	83	1	1090	197	98.81	20.42	25	7	227	87	78.13	32.37
C53529: Non-Neoplastic Disorder	1235	119	7	0	600	41	100.00	7.41	14	8	148	71	63.64	35.27
399981008: Neoplasm and/or hamartoma	4547	953	26	11	646	84	70.27	14.34	267	1	260	118	99.63	59.60
417163006: Traumatic AND/OR non-traumatic injury	9110	2065	55	8	1125	77	87.30	10.43	97	33	359	265	74.62	48.01
362975008: Degenerative disorder	1952	318	6	2	562	30	75.00	6.00	24	6	151	69	80.00	37.20
387713003: Surgical procedure	14,775	3172	97	11	1931	267	89.81	15.78	82	4	650	232	95.35	32.44
118292001: Removal	7513	2067	82	2	1231	72	97.62	11.10	104	3	336	87	97.20	36.04

*NCIt* NCI Thesaurus

**Table 6:** The precision of our previous approach introduced in [18] regarding “outside the given hierarchy” issue

Sub-hierarchy	# of All newly formalized concepts	# of Newly formalized concepts included in NCIt				# of Newly formalized concepts included in UMLS			
		# of Formalized concepts in NCIt	# of Concepts in same sub-hierarchy	# of Concepts in other sub-hierarchy of NCIt	Precison (%)	# of Validated via UMLS	# of Validated concepts with overlapping semantic types	# of Validated concepts with semantic inconsistencies	Precison (%)
C27551: Disorder by Site	9114	1250	297	953	23.76	451	251	200	55.65
C3262: Neoplasm	8511	774	302	472	39.02	289	179	110	61.94
C53529: Non-Neoplastic Disorder	1279	466	45	421	9.66	227	125	102	55.07

*NCIt* NCI Thesaurus

### Comparison with our previous work

In our previous work [18], words appearing in the concept names were considered as FCA attributes while constructing formal context. Applying multistage intersection on FCA attributes identified newly formalized bags of words for potentially missing concepts. In this case, there was no ready-to-use concept names, and when performing validation we need to enumerate all the possible sequences of words to generate different candidates for a concept name. In this work, we use concept name sequences as FCA attributes. New concepts are formalized by computing the longest common substrings between sequences. This reformulated sequence-based intersection enables the generation of ready-to-use concept names rather than unordered bags of words from our previous approach. Also, in some cases, non-consecutive shared words between two concept names could be meaningless. For instance, intersecting bag of words of “Recurrent Adult Brain Neoplasm” (C7884) and “Recurrent Childhood Brain Stem Glioma” (C9190) results in {brain, recurrent}, which does not form a piece of valid meaning. Using the sequence-based approach in this work, we can get two concepts (i.e., longest common substrings) “recurrent” and “brain” that will not be considered as potentially missing due to having no potential supertype. In fact, both concepts are outside the given hierarchy—“Recurrent” (C14173) locates in the hierarchy of *Property or Attribute* and “Brain” (C12439) is a subtype of *Body Part* in the NCI Thesaurus.

Another notable improvement of this work is that we predict the positions where the potentially missing concepts can be added. During the process, the issue of newly formalized concepts representing different fields of knowledge could be relieved. Table 6 shows how many newly generated concepts by our previous approach are actually outside the given hierarchy. For example, while auditing “Disorder by Site” sub-hierarchy, our previous approach derived 9111 concepts from the formal context, among which 1250 are included in the NCI Thesaurus. However, 953 of 1250 are included in other sub-hierarchies in NCI Thesaurus. For the potentially missing concepts validated by external terminologies in the UMLS, 200 out of 451 have no shared semantic types with their subtypes. The “outside the given hierarchy” cases account for a large proportion of the results. In contrast, the concept positioning enhancement adopted by work can greatly help filter such “outside the given hierarchy” cases and thus improve the precision of suggested missing concepts.

### Comparison with other approaches

As mentioned previously, there are mainly two types of approaches to identify missing or new concepts for biomedical terminology enrichment.

The first type mainly imports concepts from external sources. For instance, Chandar et al. developed a similarity-based method that suggested extracted phrases from text corpus as new concepts for the SNOMED CT [11]. Peng et al. analyzed connected matrices from Gene Ontology and biological network to identify new terms for Gene Ontology [12]. He et al. leveraged alignments between different ontologies to suggest new concepts for the SNOMED CT [13] and NCI Thesaurus [14]. The work in this category relies on extrinsic knowledge to suggest new concepts and to some extent ignore the sophisticated intrinsic knowledge in the terminology itself. Compared with these approaches, our FCA approach utilizes intrinsic knowledge to detect potentially missing concepts and suggest concept positions in the hierarchy. The extrinsic knowledge is leveraged for automated validation. The other type mainly utilizes the intrinsic knowledge within the ontology itself. Previously, we introduced a structural-lexical method by mining lexical patterns in non-lattice subgraphs, where one of the patterns automatically identifies missing concepts in the SNOMED CT [15]. However, since it was applied to substructures and the lexical pattern did not exist universally in the terminology, the number of missing concepts identified was limited. As a comparison, our method in this paper could be applied to the entire hierarchy (e.g., not subject to substructures and concept names could be found for every concept) and uncover more missing concepts. Jiang and Chute performed FCA on logical definitions to search for possible missing concepts in the SNOMED CT [16]. However, due to the computational limitation, their method was only applied to a small portion of SNOMED CT concepts. Zhu et al. improved Jiang and Chute’s work by developing a scalable multistage algorithm called Spark-MCA [17] that enabled an exhaustive FCA evaluation on all the SNOMED CT concepts. A limitation of these two FCA-based approaches is that the potentially missing concepts identified only involved ungrouped logical definitions from which it is difficult to come up with the concept names. Therefore, it is inconvenient to validate those missing concepts. Compared with these two previous FCA approaches, our work provides ready-to-use concept names for the detected missing concepts on which we can apply automatic validation via extrinsic knowledge.

### Potential reasons for missing concepts

**Fig. 4:**
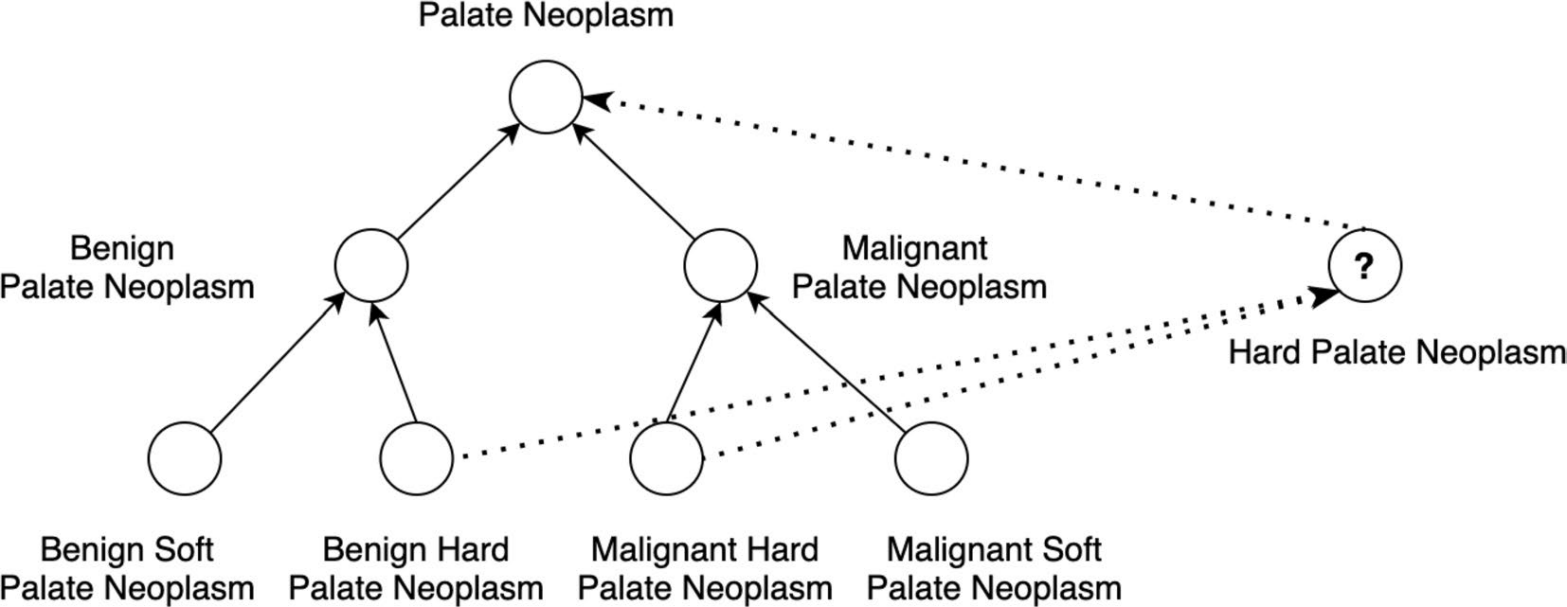
An example hierarchy generated by concept “Palate Neoplasm” (C4402) and some of its subtypes. New concept “hard palate neoplasm” could be derived by intersecting concept “Malignant Hard Palate Neoplasm” (C3528) and “Benign Hard Palate Neoplasm” (C4403), which has a potential supertype “Palate Neoplasm” (C4402)

Regarding the possible reasons leading to missing concepts in a terminology, one is that some missing concepts maybe post-coordination expression of two or more existing concepts in the terminology, designed in that way intentionally. Another cause of missing concepts maybe that certain aspects of existing domain knowledge have not been represented in the terminology yet. For instance, in Fig. 4, a potentially missing concept “Hard Palate Neoplasm” in the NCI Thesaurus could be derived by intersecting existing concepts “Malignant Hard Palate Neoplasm” (C3528) and “Benign Hard Palate Neoplasm” (C4403), which are currently classified based on if the palate neoplasm is cancerous (i.e., malignant or benign). The other way to classify them is based on the finding site: hard palate or soft palate, a missing aspect not yet modeled in the NCI Thesaurus. Note that both ways of classification are valid in SNOMED CT.

### Limitations and future work

One limitation of this work is that we only performed automatic validation of the potentially missing concepts identified via UMLS and PubMed. Incorporation of such potentially missing concepts into the respective terminologies still needs manual review and evaluation by terminology curators. Since different terminologies are developed for disparate purposes, the ways to construct the hierarchy may be different. As a result, the potentially missing concepts detected by our approach may not be directly imported due to different construction conventions. Take the above-mentioned example shown in Fig. 4, even though “Hard Palate Neoplasm” is a valid concept and its suggested position is also correct in terms of the semantic meaning, terminology curators are still required to decide whether it is necessary to include the new concept based on the classification conventions of a terminology and its target applications. Additionally, if a concept cannot be validated via external terminologies or literature, it is considered unvalidated (or no supporting evidence), and manual review by curators is also needed to determine if it is a valid missing concept for the terminology. We plan to hand over some samples of the potentially missing concepts identified and their potential positions in the hierarchies to the terminology curators (e.g., curators from the NCI Enterprise Vocabulary Service (EVS)) so that a more comprehensive evaluation of our approach could be accomplished.

Our sequence-based FCA approach is limited in a couple of ways. Firstly, our sequence-based FCA approach cannot generate new concepts whose word(s) do not appear in the vocabulary of the existing terminology. This is because we considered concept name sequences as FCA attributes, and FCA formal concepts were obtained by computing the longest common substring(s) among sequences. Secondly, we rely on the substring relation between concept names to determine if two concepts have subconcept-superconcept relation. However, there are concepts not satisfying the substring relations but having subsumption relation, such as “Carcinoma” is a descendant of “Neoplasm.” Therefore, unlike traditional FCA that formalizes the hierarchy totally based on subconcept-superconcept relations among formal concepts, in this work, we keep the original hierarchy and investigate the subconcept-superconcept relations between newly formalized concepts and original concepts to pinpoint where the potentially missing concepts may be inserted. However, our approach cannot establish connection between concepts that do not comply with the substring relation but indeed have subconcept-superconcept relations in their semantic meanings. In addition, a substring of a concept may not always be a supertype of the concept. Such cases in our result would be considered as false positives for potentially missing concepts identified by our approach.

Previously, we have studied different layouts of a concept name (e.g., breaking the concept name as a combination of noun phrases and words used in [27] and sequence representation based on sub-term and pos-tagging in [37]). In the future, we plan to utilize these variants and further define operations on those models so that more general or more detailed concepts could be generated from concept name transformation.

## Conclusions

In this paper, we introduced a sequence-based FCA approach to identifying potentially missing concepts in the NCI Thesaurus and SNOMED CT. Concept name sequences were considered as FCA attributes and ready-to-use concept names can be directly derived by computing the longest common substrings. The subconcept-superconcept relations between newly formalized concepts and original concepts were leveraged to pinpoint the location where the potentially missing concepts can be added. The automated validation via extrinsic knowledge from UMLS and PubMed showed encouraging evidence for the effectiveness of our method. Our sequence-based FCA approach for identification of potentially missing concepts is generally applicable to other terminologies.

## Data Availability

The algorithm for identifying potentially missing concepts and results are available at https://github.com/fengbozheng/BMC2021_FCA.

## Abbreviations

FCA: Formal concept analysis
NCI: National Cancer Institute
UMLS: Unified Medical Language System
NLP: Natural Language Processing
CUI: Concept Unique Identifier
OMIM: Online Mendelian Inheritance in Man
HPO: Human Phenotype Ontology
CHV: Consumer Health Vocabulary
NCIt: NCI Thesaurus

## References

[1] Bodenreider O. Biomedical ontologies in action: role in knowledge management, data integration and decision support. Yearbook of medical informatics. 2008;p. 67. PMC2592252 18660879

[2] Hoehndorf R, Schofield PN, Gkoutos GV (2015). The role of ontologies in biological and biomedical research: a functional perspective. Brief Bioinform.

[3] Bodenreider O, Burgun A (2009). Desiderata for an ontology of diseases for the annotation of biological datasets. Nat Preced.

[4] BioPortal. https://bioportal.bioontology.org/. Accessed 15 Feb 2021.

[5] Noy NF, Shah NH, Whetzel PL, Dai B, Dorf M, Griffith N (2009). BioPortal: ontologies and integrated data resources at the click of a mouse. Nucleic Acids Res.

[6] Salvadores M, Alexander PR, Musen MA, Noy NF (2013). BioPortal as a dataset of linked biomedical ontologies and terminologies in RDF. Semant Web.

[7] Cui L, Tao S, Zhang GQ (2016). Biomedical ontology quality assurance using a big data approach. ACM Trans Knowl Discov Data.

[8] Grau BC, Motik B, Stoilos G, Horrocks I (2012). Completeness guarantees for incomplete ontology reasoners: theory and practice. J Artif Intell Res.

[9] SNOMED International Release Management Home. https://confluence.ihtsdotools.org/display/RMT/. Accessed 15 Feb 2021.

[10] Overview of NCI Thesaurus. https://wiki.nci.nih.gov/pages/viewpage.action?pageId=7472532. Accessed 15 Feb 2021.

[11] Chandar P, Yaman A, Hoxha J, He Z, Weng C. Similarity-based recommendation of new concepts to a terminology. In: AMIA annual symposium proceedings, vol. 2015. American Medical Informatics Association; 2015. p. 386. PMC4765685 26958170

[12] Peng J, Wang T, Wang J, Wang Y, Chen J (2016). Extending gene ontology with gene association networks. Bioinformatics.

[13] He Z, Geller J, Chen Y (2015). A comparative analysis of the density of the SNOMED CT conceptual content for semantic harmonization. Artif Intell Med.

[14] He Z, Chen Y, de Coronado S, Piskorski K, Geller J. Topological-pattern-based recommendation of UMLS concepts for National Cancer Institute thesaurus. In: AMIA annual symposium proceedings, vol. 2016. American Medical Informatics Association; 2016. p. 618. PMC5333219 28269858

[15] Cui L, Zhu W, Tao S, Case JT, Bodenreider O, Zhang GQ (2017). Mining non-lattice subgraphs for detecting missing hierarchical relations and concepts in SNOMED CT. J Am Med Inform Assoc.

[16] Jiang G, Chute CG (2009). Auditing the semantic completeness of SNOMED CT using formal concept analysis. J Am Med Inform Assoc.

[17] Zhu W, Zhang G, Cui L. Spark-MCA: Large-scale, exhaustive formal concept analysis for evaluating the semantic completeness of SNOMED CT. In: AMIA annual symposium proceedings; 2017. p. 1914–23. PMC5977568 29854265

[18] Zheng F, Cui L. A lexical-based formal concept analysis method to identify missing concepts in the NCI Thesaurus. In: 2020 IEEE international conference on bioinformatics and biomedicine (BIBM). IEEE; 2020. p. 1757–60. 10.1109/bibm49941.2020.9313186 PMC8552537 34721941

[19] Ignatov DI. Introduction to formal concept analysis and its applications in information retrieval and related fields. In: Russian summer school in information retrieval. Springer; 2014. p. 42–141.

[20] Ganter B, Wille R. Formal concept analysis: mathematical foundations. Springer; 2012.

[21] Zweigenbaum P, Bachimont B, Bouaud J, Charlet J, Boisvieux JF (1995). Issues in the structuring and acquisition of an ontology for medical language understanding. Methods Inf Med.

[22] Lindberg DA, Humphreys BL, McCray AT (1993). The Unified Medical Language System. Methods Inf Med.

[23] Bodenreider O (2004). The Unified Medical Language System (UMLS): integrating biomedical terminology. Nucleic Acids Res.

[24] Martinez D, Otegi A, Soroa A, Agirre E (2014). Improving search over electronic health records using UMLS-based query expansion through random walks. J Biomed Inform.

[25] Aronson AR. Effective mapping of biomedical text to the UMLS Metathesaurus: the MetaMap program. In: Proceedings of the AMIA symposium. American Medical Informatics Association; 2001. p. 17. PMC2243666 11825149

[26] Adamusiak T, Shimoyama N, Shimoyama M (2014). Next generation phenotyping using the Unified Medical Language System. JMIR Med Inform.

[27] Zheng F, Shi J, Yang Y, Zheng WJ, Cui L (2020). A transformation-based method for auditing the IS-A hierarchy of biomedical terminologies in the Unified Medical Language System. J Am Med Inform Assoc.

[28] Yao L, Mao C, Luo Y (2019). Clinical text classification with rule-based features and knowledge-guided convolutional neural networks. BMC Med Inform Decis Mak.

[29] UMLS Reference Manual. https://www.ncbi.nlm.nih.gov/books/NBK9676/. Accessed 15 Feb 2021.

[30] PubMed Online Training. https://learn.nlm.nih.gov/documentation/training-packets/T0042010P/. Accessed 15 Feb 2021.

[31] LuiNorm. https://lexsrv3.nlm.nih.gov/LexSysGroup/Projects/lvg/2021/docs/userDoc/tools/luiNorm.html. Accessed 10 Jan 2021.

[32] Troy AD, Zhang GQ, Tian Y. Faster concept analysis. In: International conference on conceptual structures. Springer; 2007. p. 206–19.

[33] MEDLINE/PubMed Data Documentation. https://www.nlm.nih.gov/databases/download/pubmed_medline_documentation.html. Accessed 27 Feb 2021.

[34] Welcome to Apache Lucene. https://lucene.apache.org/. Accessed 27 Feb 2021.

[35] Doniec JM, Löhnert MS, Schniewind B, Bokelmann F, Kremer B, Grimm H (2003). Endoscopic removal of large colorectal polyps. Dis Colon Rectum.

[36] Gu GM, Epstein JB, Morton TH (2003). Intraoral melanoma: long-term follow-up and implication for dental clinicians. A case report and literature review. Oral Surg Oral Med Oral Pathol Oral Radiol Endodontol.

[37] Abeysinghe R, Hinderer EW, Moseley HN, Cui L (2020). SSIF: subsumption-based sub-term inference framework to audit gene ontology. Bioinformatics.

